# Spanish cultural adaptation and validation of the shoulder pain and disability index, and the oxford shoulder score after breast cancer surgery

**DOI:** 10.1186/s12955-015-0256-y

**Published:** 2015-05-23

**Authors:** María Torres-Lacomba, Beatriz Sánchez-Sánchez, Virginia Prieto-Gómez, Soraya Pacheco-da-Costa, María José Yuste-Sánchez, Beatriz Navarro-Brazález, Carlos Gutiérrez-Ortega

**Affiliations:** Physiotherapy Department, Physiotherapy in Women’s Health Research Group, Faculty of Medicine and Health Sciences, University of Alcalá, Alcalá de Henares, Madrid Spain; Department of Epidemiology, Medical Statistics Unit, Central de la Defensa Hospital, Madrid, Madrid Spain; Faculty of Medicine and Health Sciences-University of Alcalá, Campus Externo, Ctra. Madrid-Barcelona km 33.600, Alcalá de Henares, Madrid 28071 Spain

**Keywords:** Oxford shoulder score, Shoulder pain and disability index, Validity, Responsiveness, Breast cancer, Spanish

## Abstract

**Background:**

The Oxford Shoulder Score (OSS) and the Shoulder Pain and Disability Index (SPADI) are patient-based outcome scores with valid psychometric properties which are widely used for shoulder interventions.

**Objective:**

The purpose of the study is to adapt both questionnaires cross-culturally to Spanish, and to test their reliability, validity, responsiveness, and feasibility.

**Design:**

Cultural adaptation and psychometric validation study.

**Methods:**

Consecutive patients who had undergone breast cancer surgery referred to an outpatient clinic at the University of Alcalá de Henares, Spain. One hundred and twenty women who had undergone breast cancer surgery, with pain and shoulder dysfunction. Cross-cultural adaptation was performed according to the international guidelines. Reliability was analysed by test-retest reliability and internal consistency. Content and convergent construct validity were measured by the Expert Committee’s and Spearman coefficient respectively. Responsiveness, feasibility, floor and ceiling effects were also tested.

**Results:**

One hundred and twenty women aged 54.2 (±11) years took part in the study. The reliability was excellent; test-retest reliability was 0.974 (p < 0.001) for OSS, and 0.992 (p < 0.001) for SPADI; and Cronbach’s alpha value was 0.947 for OSS, and 0.965 for SPADI. High construct validity was found between the OSS and SPADI questionnaires (r = −0.674). The effect size (ES) and standardized response mean (SRM) was moderate in OSS (ES = 0.50 and SRM = 0.70 (p < 0.001)), and moderate to good in SPADI (ES = 0.59 and SRM = 0.82 (p < 0.001)).

**Limitations:**

This study has some limitations, such as the group of participants is composed only of women following breast cancer treatment; the measurement took place in a single centre; and all the questionnaires administered were always provided to the participants in the same order.

**Conclusions:**

The OSS and SPADI Spanish versions are applicable, reliable, valid, and responsive to assess shoulder symptoms and quality of life in Spanish women with shoulder pain and disability after breast cancer treatment.

**Electronic supplementary material:**

The online version of this article (doi:10.1186/s12955-015-0256-y) contains supplementary material, which is available to authorized users.

## Introduction

Shoulder pain and shoulder function impairment are common complaints of women treated for breast cancer (BC) that can persist for up to six years after surgery [1–3]. Following BC surgery, 25% to 60% of patients present persistent pain [4], and 35% of women experience different levels of moderate arm/shoulder pain in the first six months following breast surgery [5]. Axillary web syndrome (AWS), frozen shoulder, shoulder pain, shoulder range of motion (ROM) restriction (especially in flexion, abduction and external rotation movements), numbness, tightness and weakness, and upper-limb lymphedema are frequently related to BC surgery [6–13]. Shoulder ROM restriction is found in 21–30% of women; [6, 7] 9–68% of women complain of shoulder/arm pain [9], and up to 56% report difficulties in lifting their upper limb or reaching overhead [2, 7]. These symptoms are associated with a decrease in women’s functional status and quality of life (QoL) [2, 14], and have a negative impact in a woman’s ability to care for her family and/or return to work [15, 16]. At present, shoulder pain and disability is recognized as an important post-operative factor that affects QoL in women undergoing BC surgery [1–3, 7, 8, 15, 16].

Health-related quality of life (HRQoL) or health status are established criteria for therapeutic measures assessment [17]. Several validated instruments are available for one body region or one specific disease, especially in the English language. In the past decades, several functional scales have been developed for specific measurement of the functional impact of shoulder disorders [18]. Nevertheless, there is a need to design specific measures for use in non-English-speaking countries, because different cultural groups may vary in disease expression and health-care systems. This need has become more essential with the growing number of multicentre and multinational studies. The presence of culturally equivalent instruments would allow international comparison of national studies, simplifying the problems of meta-analysis for clinical research.

At present, the only instrument for QoL assessment related to shoulder pain which is available in Spanish is the Netherlands Shoulder Disability Questionnaire (NSDQ) [19], which was validated for the Mexican population. Therefore, there is no specific shoulder questionnaire that is validated for the Spanish population, because cultural differences may exist between the two countries.

The Oxford Shoulder Score (OSS) and the Shoulder Pain and Disability Index (SPADI) are internationally used patient-based outcome scores. The original English OSS and SPADI are easy to complete, impose very little burden on the patient and provide reliable, valid and responsive data from the patient’s perception of their shoulder problems [20–25].

The aim of the present study was to translate, to adapt culturally and to validate the original OSS and SPADI questionnaires to obtain the respective Spanish versions in accordance with internationally accepted guidelines, and to assess the validity, reliability, sensitivity to change, and feasibility of the Spanish OSS and SPADI versions.

## Methods

**Fig. 1:**
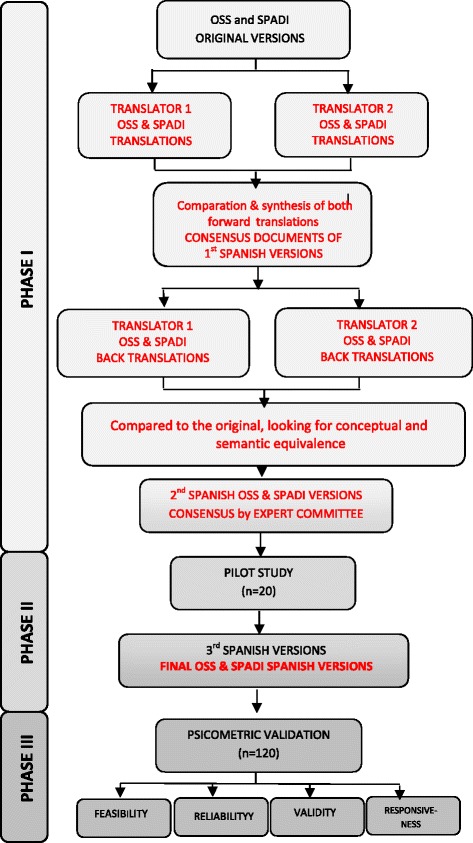
Flowchart of the translation and cultural adaptation of the OSS and SPADI

Between March 2011 and December 2013, 120 women with shoulder pain and disability during the first six months after BC surgery were consecutively recruited for this study. Subjects who had cognitive impairment, shoulder instability, neurological and rheumatologic disease, pain from chemotherapy, and with visual impairment for reading, were excluded from the study. All participants were native Spanish speakers. The study protocol was approved by “Príncipe de Asturias” University Hospital Clinical Research Ethics Committee in Alcalá de Henares, Madrid, Spain. Full informed consent was obtained from each participant prior to participation after receiving complete information on the study. The study was developed in three phases according to the ISPOR Task Force for Translation and Cultural Adaptation [26], and Isis outcomes translation and linguistic validation process (Fig. 1). Initially, the SPADI author and Isis Outcomes for OSS, were contacted to obtain their permission to conduct the study, and to ensure that concurrent studies would not be performed in parallel to this study.

### Phase I: translation and culturally adaptation process

The translation and culturally adaptation process was developed according to the ISPOR Task Force for Translation and Cultural Adaptation [26], and is explained in Fig. 1. In addition to that, SPADI’s author and Isis Outcomes were notified and agreed to the preliminary SPADI and OSS Spanish version respectively.

### Phase II: pilot testing

Both the preliminary SPADI and OSS Spanish versions were administered to 20 native Spanish-speaking women who met the inclusion criteria in order to obtain the SPADI (see Additional file 1) and OSS (see Isis Outcomes website) Spanish versions.

### Phase III: psychometric validation process

SPADI and OSS Spanish versions were tested for reliability, validity, responsiveness and feasibility in 120 women after BC treatment. Sample size was based on the general recommendations by Altman et al. [27] and Terwee [28] who stated that at least 50 subjects would be recommended for the assessment of measurement. Besides, Gorsuch [29] and Kline [30] affirm that sample size should not be less than 100 subjects, even though the number of variables is less than 20; and on Bryant & Yarnold [31] of the subjects-to-variables ratio should be no lower than 5 [32]. Women’s socio demographic and clinical history data were recorded. In order to analyse the convergent construct validity, the participants filled the SPADI and OSS Spanish versions with the following questionnaires: the Functional Assessment of Cancer Therapy-Breast (FACT-B) [33], the Netherlands Shoulder Disability Questionnaire (NSDQ) [34], and the SF-36 Health Survey [35].

### Questionnaires

The SPADI is a self-report questionnaire that measures shoulder pain and disability. It consists of 13 items in two subscales: pain (5 items) and disability (8 items). The items of both subscales are graded from 0 (no pain or disability) to 10 (the worst imaginable pain or so much difficulty on performing tasks that help is required). The higher the score in each subscale the higher the pain intensity and the greater the disability. To obtain a total score for the SPADI, the pain and disability subscale scores are averaged [23].

**OSS** is a condition-specific self-report questionnaire developed for patients who have shoulder dysfunction other than instability. It contains 12 items about pain and disability to be answered by the patient independently. There are five categories of responses for every question, corresponding to a score ranging from 0 to 4, with 4 representing the best, according to the new scoring system. Scores are combined to give a single score, with a range from 0 (most severe symptoms) to 48 points (least symptoms), so that the lower scores indicate more pain and disability [21]. All respondents are asked to consider how their shoulder has felt for the last four weeks when completing the questionnaire [20].

**FACT-B** is a 37-item self-administered questionnaire designed to measure multidimensional QoL in patients with BC. It consists of FACT-General (FACT-G) plus the BC Subscale (BCS), which complements the general scale with items specific to QoL in BC. The assessment is comprised of five domains (physical well-being (PWB), social/family well-being (SWB), emotional well-being (EWB), functional well-being (FWB), and BCS). Each item is rated on a five-point Likert scale. FACT-B total score is the sum of the scores of all five subscales, and can range from 0 to 136. The trial outcome index (TOI) is the sum of the PWB, FWB, and BCS scores, with scores in the range of 0–84. Because the FACT-G includes some items that are worded positively, the responses to all negative items were reversed for all analysis. A higher score therefore represents better HRQoL [36].

**NSDQ** is a pain-related disability questionnaire designed for self-administration. It includes 16 items describing common situations that may induce symptoms in patients with shoulder disorders, all referring to the preceding 24 hours. Response options are either ‘yes’, ‘no’, or ‘not applicable’. A final score is calculated by dividing the number of positively scored items by the total number of applicable items, and subsequently multiplying the score by 100, resulting in a final score ranging from 0 (no disability) and 100 (all applicable items positive). The higher the score the greater the disability [34].

**SF-36** Health Survey is a 36-item questionnaire widely used to assess general health. It provides scores on eight dimensions: physical function, social function, limitations caused by physical symptoms, limitations caused by emotional problems, general mental health, vitality, pain, and perception of general pain. Scores for each dimension range from 0 (poor health) to 100 (good health) [35].

### Reliability

Reliability was assessed by internal consistency which is determined by the degree to which all items measure the same thing, measured using Cronbach’s alpha (α), which ranges from 0 to 1. Values greater than 0.7 shows good reliability (ranges from 0.7 and 0.9); although 0.6 may be acceptable. The higher the value, the greater the internal consistency [37]. Reliability was also assessed by test-retest, which was assessed by asking 20 participants to complete another OSS and SPADI 48 hours after the first test. The change in mean scores between the test-retest reliability was evaluated by the intraclass correlation coefficient (ICC), the values of which are from 0 (no stability) to 1 (perfect stability) [38].

### Validity

Validity identifies the extent to which an instrument measures what it is designed for. This was assessed through content, face and convergent construct validity. Although content validity was ensured by the development of the original scale, the Expert Committee’s composed of a methodologist, 3 health professionals (1 physical therapist, 1 physician, 1 gynaecologist), 1 language professional and 2 translators (forward and backward translators); face validity was ensured by the pilot study subjects’ opinion. Content and face validity were taken into account to validate the ability of items to collect the health status of respondents. Convergent construct validity was measured with a multiple comparison with questionnaires that are mainly used for shoulder pain and dysfunction assessment, assuming that correlations and mean comparisons between groups of participants with versions of validated questionnaires would run, in all cases, in the right direction. Therefore, the correlation between the SPADI and OSS Spanish versions was calculated with FACT-B, NSDQ and SF-36 adapted and validated for the Spanish population. Convergent construct validity was evaluated using Spearman’s correlation (r), high validity being considered when the range was between 0.30 and 0.40.

### Responsiveness

In order to determine responsiveness, or sensitivity to change, a standard Physiotherapy program for shoulder pain and dysfunction was developed in women who participated in the study. The program consisted of a three-week period with three visits per week [11–13]. Patients filled the questionnaires again during their final physical therapy session. Pre- and post-Physiotherapy intervention scores (dimensions and totals) were calculated by comparing statistical tests for paired data scores for each of the questionnaires provided: SPADI, OSS, FACT-B, NSDQ and SF-36. P-values, effect size (ES) and standardized response mean (SRM) were calculated to evaluate these questionnaires responsiveness. P-value was calculated using the Wilcoxon signed-rank test comparing pre- and post-treatment score. ES is the mean change in the score divided by the standard deviation of individuals baseline score, and the SRM is the mean change in the score divided by the standard deviation of individual changes in score. For both values, ES and SRM, values between 0.20 and 0.50 are considered as small sensitivity, 0.50 and 0.80 as moderate sensitivity, 0.80 and 1 as good sensitivity and over 1 excellent. These statistics are analysed not only to validate the SPADI and OSS questionnaires, but also the rest of the questionnaires provided.

### Ceiling and floor effects, and feasibility

Possible ceiling and floor effects were analysed for individual items and total scores for both questionnaires, with such effects considered as being present if more than 15% of respondents achieved the highest or lowest score, respectively [39]. The feasibility was assessed by the average administration time.

A *P*-value of <0.05 was considered statistically significant.

For statistical analysis of the data obtained from the questionnaires, SPSS® version 15 for Windows® was used.

## Results

The translation and cultural adaptation of the SPADI and OSS revealed no difficulties. In some items the translators used different synonyms or minimal discernible differences. However, a unanimous consensus was obtained to ensure final versions applicable to the Spanish culture. The conceptual and linguistic equivalence was retained for the SPADI and OSS Spanish versions. Between March 2011 and December 2013, 120 women with pain and shoulder dysfunction were consecutively recruited for the psychometric validation. Socio-demographics and clinical characteristics are shown in Table 1.

**Table 1:** Socio-demographics and clinical characteristics

Age (years) Mean (SD)	54.2(11)
Body mass index Mean (SD)	27.1(5.7)
**Civil status** (Number (%))	
Married/partnership	106(88.3)
Single/widowed/divorced	12(10)
Unknown	2(1.7)
**Education** (Number (%))	
Primary	58(48.3)
Secondary/professional	40(33.3)
Higher	18(15)
Unknown	4(3.3)
**Socio economic level** (Number (%))	
<12.000 €	37(30.8)
12.000-24.000 €	43 (35.8)
24.000-36.000 €	15(12.5)
36.000-48.000 €	3(2.5)
>48.000 €	2(1.7)
Unknown	20(16.7)
**Surgical side** (Number (%))	
Right	60(50)
Left	57(47.5)
Bilateral	3(2.5)
**Surgical procedure** (Number (%))	
Lumpectomy	43(35.8)
Quadrantectomy	22(18.3)
Modified mastectomy	55(45.8)
Lymphadenectomy	104(86.6)
**Adjuvant therapy **(Number (%))	
Chemotherapy	99(82.5)
Radiotherapy	112(93.3)
Hormonal therapy	87(72.5)
**Sequelae** (Number (%))	
Axillary web syndrome	71(59.1)
Seroma	14(11.6)
Myofascial pain syndrome	34(40.8)

### Reliability

Concerning reliability, internal consistency and test-retest showed high rates (Table 2). Cronbach’s α coefficient for the SPADI was 0.965 and its subscales were 0.931 for the pain subscale, and 0.953 for the disability subscale. For OSS, Cronbach’s α was 0.947. The ICC was 0.992 for the SPADI and 0.974 for the OSS. Therefore, test-retest reliability was excellent in both cases. All the values were statistically significant (p < 0.001).

**Table 2:** Internal consistency and test-retest reliability for OSS and SPADI and subscales

	Test-retest				Internal consistency	
	(n = 20)				(n = 120)	
	Test	Retest	ICC	*P-*value	Cronbach’s α	*P*-value
	Md (IQR)	Md (IQR)		(for ICC)		(for Cronbach’s α)
**SPADI**	18.1(31.73)	18.85(30.77)	0.992	<0.001	0.965	<0.001
Pain subscale	19(42)	19(36)	0.986	<0.001	0.931	<0.001
Disability subscale	17.5(22.81)	19.38(26.25)	0.991	<0.001	0.953	<0.001
**OSS**	37.5(10.75)	38(8.75)	0.974	<0.001	0.947	<0.001

Md: Median; IQR: Interquartile range; ICC: Interclass Correlation Coefficients

### Validity

The Expert Committee and pilot study subjects’ reviews assessed and confirmed the content validity. Regarding convergent construct validity, Spearman’s rank correlation matrix of the SPADI and OSS with the FACT-B, NSDQ and SF-36 are shown in Table 3. Construct validity was considered high between the OSS and SPADI questionnaires with a negative correlation (r = −0.674). The Spearman’s rank of the OSS questionnaire with each SPADI dimension, were negative: pain scale r = −0.640 and disability scale r = −0.645. In all cases, P-value was <0.01. Regarding the SPADI and OSS correlations with the other questionnaires, both showed good values in correlations with: NSDQ (r = 0.432 and −0.469), SF-36 physical function dimension (r = −0.452 and 0.364), SF-36 physical role dimension (r = −0.392 and 0.391), and SF-36 bodily pain dimension (r = −0.385 and 0.363), and SF-36 emotional role dimension (r = −0.315 and 0.312) (with the SPADI and OSS respectively) in all cases with a P-value <0.01. The total SPADI and its pain scale also show a good relation with the FACT-B questionnaire (r = −0.298 and −0.343 respectively). This way, score of SPADI and its dimensions showed better values with TOI dimension of FACT-B (r = −0,404, −0,448 and −0,340) with a P-value <0.01.

**Table 3:** Spearman’s coefficient (r) of convergent construct validity

	SPADI	SPADI-pain subscale	SPADI-disability subscale	OSS	Fact-B	Fact-G	TOI	NSDQ	SF-36- physical function	SF-36- physical role	SF-36- bodily pain	SF-36- general health	SF-36-vitality	SF-36- social role	SF-36- emotional role	SF-36- mental health
**SPADI**	-															
Pain subscale	0.931**	-														
Disability subscale	0.970**	0.827**	-													
**OSS**	−0.674**	−0.640**	−0.645**	-												
Fact-B	−0.298*	−0.343**	−0.247**	0.213*	-											
Fact-G	−0.208*	−0.235*	−0.179	0.114	0.871**	-										
TOI	−0.404**	−0.448**	−0.340**	0.276**	0.923**	0.711**	-									
NSDQ	0.432**	0.436**	0.396**	−0.469**	−0.255**	−0.191*	−0.317**	-								
SF-36-physical function	−0.452**	−0.463**	−0.438**	0.364**	0.164	0.079	0.226*	−0.292**	-							
SF-36-physical role	−0.392**	−0.405**	−0.364**	0.391**	0.175	0.182	0.236*	−0.285**	0.400**	-						
SF-36- bodily pain	−0.385**	−0.355**	−0.377**	0.363**	0.139	0.057	0.191*	−0.087	0.310**	0.477**	-					
SF-36- general health	−0.182*	−0.167	−0.202*	0.140	0.233*	0.200*	0.213*	−0.218*	0.195*	0.160	0.219*	-				
SF-36-vitality	−0.146	−0.145	−0.150	0.101	0.104	0.025	0.140	−0.071	0.220*	0.213*	0.290**	0.370**	-			
SF-36-social role	−0.200*	−0.181*	−0.200*	0.186*	0.126	0.117	0.174	−0.272**	0.247**	0.420**	0.439**	0.222*	0.503**	-		
SF-36-emotional role	−0.315**	−0.312**	−0.301**	0.312**	0.119	0.099	0.161	−0.266**	0.220*	0.494**	0.348**	0.186*	0.394**	0.417**	-	
SF-36- mental health	−0.146	−0.077	−0.177	0.051	0.049	0.054	0.102	−0.026	−0.033	0.147	0.182	0.216*	0.601**	0.511**	0.398**	-

** p < 0.01; * *p* < 0.05

The SPADI correlations and its dimensions were in all cases negative except with the NSDQ. On the other hand, the NSDQ correlations and its dimensions were in all cases negative, except with the SPADI questionnaire and its dimensions.

### Responsiveness

Responsiveness was evaluated in 118 women, because during the Physical Therapy intervention two women dropped out of the study due to family problems. Responsiveness was determined using the Wilcoxon signed-rank test comparing the pre- and post-treatment scores, ES and SRM (Table 4).

**Table 4:** Responsiveness of SPADI and OSS questionnaires

	Pre-treatment score	Post-treatment score	Mean change score	Effect size (ES)	Standardised response mean (SRM)	*P-*value
	X (SD)	X (SD)	X (SD)			
**SPADI**	32.14(25.16)	17.28(19.66)	14.86 (18.03)	0.59	0.82	<0.001
Pain subscale	33.58(25.71)	12.40(13.59)	21.18 (18.69)	0.82	1.13	<0.001
Disability subscale	31.25(26.39)	15.68(19.80)	15.57 (19.85)	0.59	0.78	<0.001
**OSS**	36.63(11.11)	41.19(8.07)	−5.56 (7.95)	−0.50	−0.70	<0.001

**SPADI:** Shoulder and Pain Disability Index; **OSS**: Oxford Shoulder Score; X (SD): Mean (Standard Deviation)

The OSS and the SPADI questionnaires and their domains were significantly improved after Physical Therapy treatment, with P-values <0.001 in all these cases. The responsiveness was moderate in the OSS, and between moderate (ES) and good (SRM) in the SPADI, the pain subscale SRM of the SPADI was excellent. The OSS questionnaire demonstrated moderate responsiveness with an ES of −0.50 and an SRM of −0.70. The SPADI questionnaire and its domains demonstrated moderate to excellent responsiveness; the disability subscale ES was 0.59 and the SRM 0.78; the pain subscale ES was 0.82 and the SRM 1.13, the latter showing excellent value. The responsiveness of the other questionnaires can be seen in Table 4.

### Ceiling and floor effects, and feasibility

No ceiling or floor effect was detected in total or item scores in either of the two questionnaires. The average time for questionnaire administration was 3.4 (±1.4) minutes for the OSS and 3 (±1.9) minutes for the SPADI.

## Discussion

There are some questionnaires for the assessment of patients with shoulder dysfunction, but none of them are validated in the Spanish population. Furthermore, the OSS and SPADI questionnaires are the most internationally used patient-based outcome scores and their original English versions are easy to fill in, reliable, valid and responsive to patient perceptions of shoulder problems. The structure of the questions is simple and easily understood, resulting in a high percentage of answers and a very good acceptance by patients, who don’t need supplementary instructions in order to answer the questions independently. The five-point Likert system enables quick answering by the patients and a very simple and quick assessment by the researcher, offering an advantage for daily clinical practice. It is critical to employ valid and reliable research measures but they must also be both culturally and linguistically appropriate. Both questionnaires have been adapted and validated to other countries, such as Germany, Italy, The Netherlands, Norway, Turkey, Slovenia, Brazil, Denmark, Korea, and Arabia [38–50].

As said before, shoulder pain and dysfunction are common problems for women who have been treated for BC, especially following surgery [4, 5]. In a qualitative systematic review published in 2014, the use of the Disabilities of Arm, Shoulder and Hand (DASH) questionnaire is recommended for this population [51], although the results should be interpreted with caution, as most studies had limitations such as small sample sizes and secondary problems like lymphedema. In fact, currently there is a specific scale for assessing the QoL in patients with lymphedema (ULL27) [52]. Therefore, there is still a need of different measurement instruments for shoulder pain and dysfunction in women who have been treated for BC, especially in the case of the present study where the participants were included in the first 6 months after surgery, when the problems are mostly related to the shoulder and not the entire upper limb.

### Translation and cultural adaptation

The development of a cultural adaptation from the OSS and SPADI through contact with the authors of the original versions, and of a rigorous compliance with recognized international guidelines and with methodology suggested by ISIS Outcome, assured a good correlation between the Spanish versions and the original English versions. The OSS and SPADI Spanish-version translations and cultural adaptation did not present any difficulties. In the case of the SPADI questionnaire, the weight expression ‘10 pounds’ was replaced with ‘5 kg’ since the metric system is used in Spain, and the same was done in the translation of the SPADI for German and Brazilian Portuguese [44, 45].

As has been remarked on by Bumin et al. in the SPADI Turkish version, the depth of interviews performed to assess the comprehensibility of the questionnaire, revealed that there is a gender-biased question (i.e. How much difficulty do you have removing something from your back pocket?), because men usually carry items in their back pocket but women generally do not [38]. Therefore, in the SPADI Spanish version this bias was also highlighted, since in this study 100% of participants were women. However, we did not adapt this item as we consider this should be analysed and, if necessary, changed by the author of the SPADI questionnaire.

### Reliability

The psychometric properties of the OSS and SPADI Spanish versions showed good internal consistence as well as those reported for the OSS and SPADI original English versions [20, 23].

Regarding internal consistency, the OSS Spanish version’s global scores are slightly higher than the ones found in the English, Danish, Dutch, Korean and Turkish versions [20, 40, 42, 50]. The SPADI Spanish version’s global scores are also slightly higher than the ones found in the English original version [22–25, 53–55] and in all the cross-cultural adapted versions [18, 38, 41, 44, 45, 47, 48] (Table 5). This fact may be due to demographic and clinical data (the present study was accomplished among women following breast cancer surgery), and geographic, cultural and health-care system differences that seem to affect QoL perception [56].

**Table 5:** Test-retest reliability and internal consistency of Spanish SPADI and OSS versions, and previous studies

Shoulder and pain disability index							
Studies		Test-retest reliability (ICC)			Internal consistency		
		Pain subscale	Disability subscale	Total	Pain subscale	Disability subscale	Total
Present study		0.98	0.99	0.99	0.93	0.95	0.96
Roach et al. [23]	1991	0.64	0.64	0.66	0.88	0.87	0.89
Beaton & Richard [53]	1998	-	-	0.91	-	-	-
Heald et al. [54]	1997	-	-	-	0.89	0.95	0.96
Roddey et al. [25]	2000	-	-	-	0.89	0.95	0.96
Schmitt & di Fabbio [55]	2004	-	-	0.86	-	-	-
MacDermid et al. [22]	2006	-	-	-	0.92	0.93	0.95
Angst et al. [46]	2007	0.89	0.93	0.94	0.92	0.93	0.95
Ekeberg et al. [24]	2008	0.72	0.85	0.85	0.74	0.89	0.91
Bumin et al. [38]	2008	0.83	0.83	-	-	-	-
Jamnik & Spevak [44]	2008	0.89	0.95	0.94	0.78	0.90	0.92
Guermazi et al. [47]	2011	-	-	0.91	-	-	0.96
Martins et al. [45]	2011	0.94	0.90	0.94	0.88	0.87	0.89
Christiansen et al. [41]	2012	0.88	0.84	0.88	0.85	0.93	0.94
Marchese et al. [48]	2012	-	-	0.91	-	-	0.89
**Oxford Shoulder Score**							
		**Test-retest reliability (ICC)**			**Internal consistency**		
Present study		0.97			0.94		
Dawson et al. [20]	1996	-			0.89 Pre-operative; 0.92 Post-operative		
Huber et al. [43]	2004	0.98			0.94		
Berendes et al. [40]	2010	0.98			0.92		
Murena et al. [49]	2010	0.97			0.95		
Tuğay et al. [39]	2011	0.97			0.92		
Frich et al. [42]	2011	0.98			0.93		
Roh et al. [50]	2012	0.95			0.91		
ICC: interclass correlation coefficient							

Concerning test-retest; the 48-hour interval was chosen taking into account the nature of the women’s shoulder morbidity in order to minimize changes in their clinical status. Pain and shoulder disability in women treated for BC may be due to different sequelae of both medical and surgical treatment of BC. Symptoms such as myofascial pain origin, axillary web syndrome, etc., are susceptible to change very quickly, depending on the cause of pain and disability [11, 12]. Other validation studies used an interval between from 1 to 4 days for OSS, and from 2 to 7 days for SPADI. The value of ICC for OSS in the present study was in accordance to the others versions. The value of ICC for SPADI in the present study was higher than those of the other versions. These differences should be related to demographic and clinical differences between the study populations. Besides, the other validity studies populations were mostly males with musculoskeletal alterations while the population of the present study was specifically female breast cancer survivors.

### Validity

This study showed a good convergent validity of the OSS and SPADI (and its dimensions) Spanish versions with the NSDQ questionnaire, and with the physical functioning, physical role, bodily pain and emotional role of the SF36. Also the total SPADI showed good values with the FACT-B and specially with the TOI dimension. We can find the best values between the SPADI and OSS questionnaires. Regarding the SPADI and NSDQ questionnaires, the correlations with other questionnaires were negative because the methods of scoring are opposite to each other. All these correlations are justified, so both, SPADI and OSS questionnaires, measure the same symptoms: shoulder pain and dysfunction, and the others instruments or dimensions measure also physical or pain aspects, or in the case of NSDQ, like these symptoms affect to common daily situations. Also the good values with the SF-36-emotional role, could be related to the emotional status of breast cancer survivors [57, 58].

### Responsiveness

The responsiveness of the OSS and SPADI Spanish versions showed lower values than those reported for the original OSS and SPADI English original versions. This fact may be due to clinical data; the present study was accomplished among women, following breast cancer surgery, with shoulder pain and disability, and the original versions were accomplished among orthopedic patients (capsulitis adhesive, impingement syndrome, rotator cuff rupture, etc.) [20, 23, 54, 59, 60].

As far as the authors are concerned, this is the first cultural adaptation study that includes the analysis of responsiveness in the OSS questionnaire. Regarding the SPADI questionnaire, this is the second cultural adaptation to include the analysis of responsiveness. The first was the Slovene version. Although the responsiveness of the Slovene version is higher than that of the present study, they tested it only with the patients who improved in terms of self-perceived severity of disability (16 patients) rather than for the entire sample [44].

### Ceiling and floor effects, and feasibility

With regards to floor and ceiling effects, the results are consistent with other studies that have analysed these effects [20, 23, 39, 41].

Concerning feasibility, OSS and SPADI Spanish versions were fully filled in and were accepted and easily completed by all the participants. No single item was responsible for non-completion of the questionnaires. The short time required to complete the questionnaires agrees with other studies [42, 43, 49] and it is slightly longer than the Turkish and Korean versions [39, 50] This suggests that the Spanish OSS and SPADI are well understood by patients whose mother tongue is Spanish.

### Limitations

This study has some limitations, such as the group of participants is composed only of women following breast cancer treatment; the measurement took place in a single centre; and all the questionnaires administered were always provided to the participants in the same order.

## Conclusions

The Oxford Shoulder Score (OSS) and the Shoulder Pain and Disability Index (SPADI) Spanish versions showed semantic, conceptual, idiomatic and content equivalence with the original versions. Both instruments are applicable, reliable, valid, and responsive for assessing shoulder symptoms and quality of life in Spanish women with shoulder pain and disability after breast cancer treatment. Consequently, both questionnaires may be useful in Spanish-speaking populations and for making cross-ethnic and –cultural comparisons with other English-speaking countries that have a large Spanish-speaking population.

## Additional file

Additional file 1:
**Shoulder pain and disability index (SPADI) Spanish version Escala de Dolor y discapacidad de hombro.**

## Abbreviations

AWS: Axillary web syndrome
BC: Breast cancer
BCS: Breast cancer subscale
DASH: Disabilities of arm, shoulder and hand
ES: Effect size
EWB: Emotional well-being
FACT-B: Functional assessment of cancer therapy-breast
FACT-G: Functional assessment of cancer therapy
FWB: Functional well-being
HRQoL: Health-related quality of life
ICC: Intraclass correlation coefficient
OSS: Oxford shoulder score
PWB: Physical well-being
QoL: Quality of life
ROM: Range of motion
SPADI: Shoulder pain and disability index
SRM: Standardized response mean
SWB: Social/family well-being
TOI: Trial outcome index
