# Supplementary material for: Spanish cultural adaptation and validation of the shoulder pain and disability index, and the oxford shoulder score after breast cancer surgery
Source: Health Qual Life Outcomes. 2015 May 23;13:63. doi: 10.1186/s12955-015-0256-y (PMC4489002; doi:10.1186/s12955-015-0256-y)
Supplement: Additional file 1: — Shoulder pain and disability index (SPADI) Spanish version Escala de Dolor y discapacidad de hombro. [file 12955_2015_256_MOESM1_ESM.pdf]

SHOULDER PAIN AND DISABILITY INDEX (SPADI) SPANISH VERSION

ESCALA DE DOLOR Y DISCAPACIDAD DE HOMBRO

Nombre del paciente ________________________________ Fecha ________________

Por favor, léalo con atención:

Instrucciones: Por favor, rodeé con un círculo el número que mejor describa la respuesta a la pregunta que se le formula.

Escala de Dolor:

Ningún dolor 0 1 2 3 4 5 6 7 8 9 10 El peor dolor imaginable

¿Cómo de severo es su dolor?

1. Su peor dolor
   0 1 2 3 4 5 6 7 8 9 10

2. Cuando está tumbado/a sobre el lado afecto
   0 1 2 3 4 5 6 7 8 9 10

3. Cuando coge algo de un estante alto
   0 1 2 3 4 5 6 7 8 9 10

4. Cuando se toca la zona posterior del cuello
   0 1 2 3 4 5 6 7 8 9 10

5. Cuando empuja con el brazo afecto
   0 1 2 3 4 5 6 7 8 9 10

Escala de Discapacidad:
Sin dificultad 0 1 2 3 4 5 6 7 8 9 10 Tan difícil que necesita ayuda

¿Cuánta dificultad tiene usted?

1. Cando se lava el pelo
   0 1 2 3 4 5 6 7 8 9 10

2. Cuando se lava la espalda
   0 1 2 3 4 5 6 7 8 9 10

3. Cuando se pone una camiseta o jersey

0	1	2	3	4	5	6	7	8	9	10

4. Cuando se pone una camisa abotonada por delante

0	1	2	3	4	5	6	7	8	9	10

5. Cuando se pone unos pantalones

0	1	2	3	4	5	6	7	8	9	10

6. Cuando coloca un objeto en un estante alto

0	1	2	3	4	5	6	7	8	9	10

7. Cuando lleva un objeto pesado de 4 kilos y medio

0	1	2	3	4	5	6	7	8	9	10

8. Cuando coge algo de su bolsillo trasero

0	1	2	3	4	5	6	7	8	9	10

OTROS COMENTARIOS:

Evaluador:
